# Phosphorylation of IWS1 by AKT maintains liposarcoma tumor heterogeneity through preservation of cancer stem cell phenotypes and mesenchymal-epithelial plasticity

**DOI:** 10.1038/s41389-023-00469-z

**Published:** 2023-05-26

**Authors:** Yu Wang, Hongji Zhang, Alessandro La Ferlita, Nipin Sp, Marina Goryunova, Patricia Sarchet, Zhiwei Hu, Michael Sorkin, Alex Kim, Hai Huang, Hua Zhu, Allan Tsung, Raphael E. Pollock, Joal D. Beane

**Affiliations:** 1 Department of Surgery, Division of Surgical Oncology, James Cancer Center, The Ohio State University, Columbus, OH USA; 2 Institute of Pathology, Tongji Hospital, Tongji Medical College, Huazhong University of Science and Technology, Wuhan, People’s Republic of China; 3 Department of Cancer Biology and Genetics, James Cancer Center, The Ohio State University, Columbus, OH USA; 4 Department of Plastic and Reconstructive Surgery, James Cancer Center, The Ohio State University, Columbus, OH USA; 5 Department of Surgery, Division of Cardiac Surgery, Wexner Medical Center, The Ohio State University, Columbus, OH USA

**Keywords:** Sarcoma, Cancer microenvironment

## Abstract

Chemotherapy remains the mainstay of treatment for patients with advanced liposarcoma (LPS), but response rates are only 25% and the overall survival at 5 years is dismal at 20–34%. Translation of other therapies have not been successful and there has been no significant improvement in prognosis for nearly 20 years. The aberrant activation of the phosphatidylinositol 3-kinase (PI3K)/AKT pathway has been implicated in the aggressive clinical behavior LPS and in resistance to chemotherapy, but the precise mechanism remains elusive and efforts to target AKT clinically have failed. Here we show that the AKT-mediated phosphorylation of the transcription elongation factor IWS1, promotes the maintenance of cancer stem cells in both cell and xenograft models of LPS. In addition, phosphorylation of IWS1 by AKT contributes to a “metastable” cell phenotype, characterized by mesenchymal/epithelial plasticity. The expression of phosphorylated IWS1 also promotes anchorage-dependent and independent growth, cell migration, invasion, and tumor metastasis. In patients with LPS, IWS1 expression is associated with reduced overall survival, increased frequency of recurrence, and shorter time to relapse after resection. These findings indicate that IWS1-mediated transcription elongation is an important regulator of human LPS pathobiology in an AKT-dependent manner and implicate IWS1 as an important molecular target to treat LPS.

## Introduction

While rare, retroperitoneal liposarcomas (LPS) are locally aggressive tumors that present significant clinical challenges and are associated with a dismal prognosis [1, 2]. Surgical resection remains the only chance for long-term survival [3, 4]. Yet, tumor recurrence occurs in most patients [4, 5]. Doxorubicin-based chemotherapy remains the mainstay of treatment for patients with unresectable LPS, but response rates are only 25%, and the 5-year survival is only 20–30% [6, 7]. Mutations driving LPS are known, but therapies directed toward specific targets have yielded results far below expectations [5, 8]. The development of novel therapies has been limited, and there has been no significant improvement in prognosis for nearly 20 years. Therefore, the identification of novel drivers of tumorigenesis may help identify novel targets for therapy remains an important unmet need [1, 8].

A major challenge hampering the development of effective LPS treatments is tumor heterogeneity and the remarkable plasticity of LPS cancer cells [9–11]. While LPS are mesenchymal tumors, recent evidence suggests that sarcoma cells may exhibit a “metastable” phenotype, characterized by the transition of tumor cells between mesenchymal and epithelial-like states [12, 13]. This plasticity appears to be a feature of tumors with more aggressive biology, as their cells can undergo mesenchymal to epithelial transition (MET), giving rise to highly proliferative cells in the primary tumor, or epithelial to mesenchymal transition (EMT), giving rise to migratory, invasive, and metastatic cells. Recent evidence also suggests a strong link between EMT/MET and the maintenance of cancer stem cells (CSC) [12, 14–16]. The EMT/MET signaling programs confer CSC-like characteristics to cells within the tumor, thereby increasing tumor heterogeneity, which has been linked to therapeutic resistance in many different types of cancer including LPS [17–19]. A more comprehensive understanding of the drivers of these programs in LPS may help develop strategies to reduce the resistance of LPS to currently available therapies.

Aberrant activation of the PI3K/AKT pathway has long been implicated in the aggressive clinical behavior of LPS and was recently shown to contribute to the self-renewal of LPS CSCs [20–23]. Specifically, pharmacologic inhibition and genetic knockdown of AKT1 and AKT2 in the human dedifferentiated LPS cell line, DDLS8817, decreased *Nanog* levels and spheroid formation in suspension cultures and reversed resistance to doxorubicin treatment and radiation therapy [22]. These findings are consistent with a previous report showing that inhibition of the PI3K/AKT pathway reduces expression of EMT markers and the abundance of CSCs in prostate cancer cell lines, rendering them more sensitive to irradiation [24]. PI3K/AKT1 signaling is also required for transforming growth factor beta-induced EMT in breast cancer, and for the maintenance of EMT in pancreatic cancer [25, 26]. While these findings highlight the pivotal role of AKT in driving oncogenesis and chemoresistance by promoting EMT and the maintenance of CSCs, efforts to target the PI3K/AKT pathway have been met with limited success (NCT02987959, NCT01048723). To date, no PI3K/AKT inhibitor has been tested in a phase III clinical trial.

Recently, AKT has been shown to promote tumor growth through activating the transcription elongation factor IWS1 [27]. IWS1 functions to recruit cofactors to the RNA polymerase II (RNAPII) complex to regulate mRNA alternative splicing [28–30]. The processing of mRNA precursors is highly regulated and occurs before the translation of almost all eukaryotic genes [31–33]. By modulating RNA splicing, IWS1 could generate oncoproteins that promote LPS [34–37]. Phosphorylation of IWS1 at Ser 720/Thr 721 by AKT, recruits the Histone H3K36 trimethyl transferase SETD2 to the SPT6/IWS1 CTD complex. Following its recruitment, SETD2 trimethylates histone H3 at K36 in the body of target genes during transcriptional elongation, catalyzing multiple steps in RNA processing [27, 28, 38]. These activities of IWS1 promote the aggressiveness of lung cancer cells [27]. Other studies have shown that IWS1 is a regulator of embryonic development and is required for the early stages of embryogenesis in mice [39], where it is required for Nanog expression [28]. Nanog is a transcription factor that plays a critical role in maintenance of CSCs in LPS and has been shown to confer LPS resistance to both chemotherapy and radiation therapy [22, 28]. Understanding how the phosphorylation of IWS1 by AKT contributes to LPS tumor biology, could identify novel AKT-dependent targets whose inhibition may materialize the unfulfilled promise of AKT inhibition.

## Results

### The AKT/IWS1 axis is active in human liposarcoma and associated with a worse prognosis in patients with liposarcoma

**Fig. 1:**
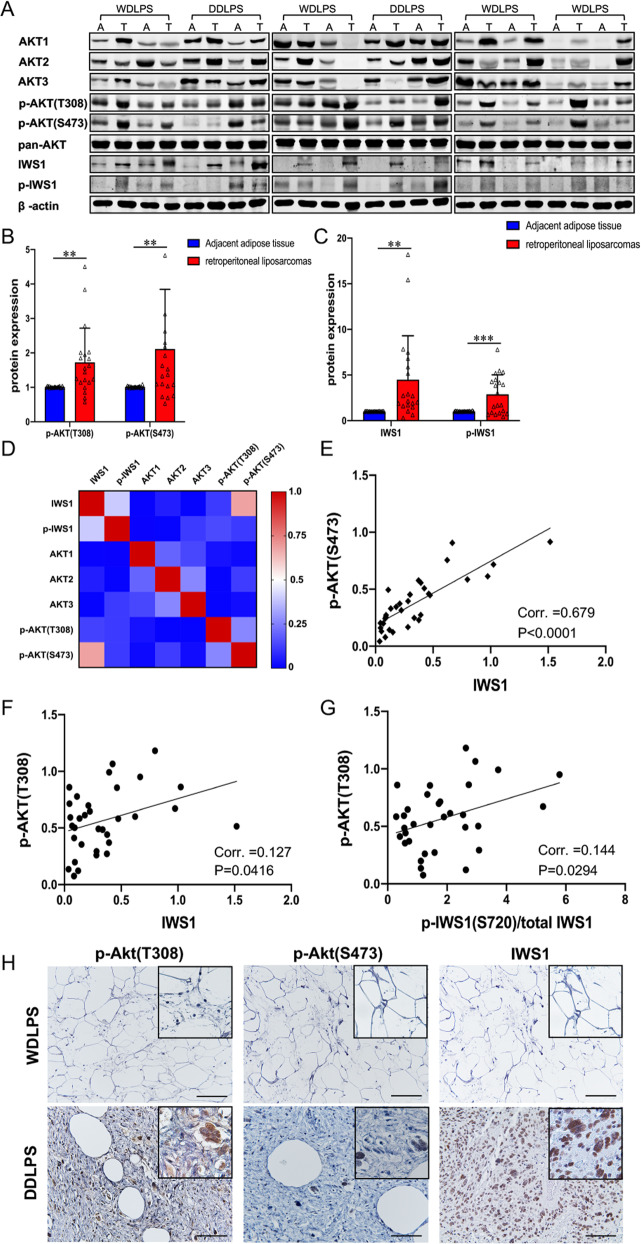
AKT/IWS1 axis is active in human LPS and associates with poor prognosis. **A** Western blot of AKT1, AKT2, AKT3, phospho-AKT (Thr 308), phospho-AKT (Ser 473), IWS1 and phospho-IWS1 in LPS tumor tissues and adjacent adipose tissues. **B** Western blot analysis the expression of phospho-AKT (Thr 308) and phospho-AKT (Ser 473) in LPS tumor tissues and adjacent adipose tissues. **C** Western blot analysis the expression of IWS1 and phospho-IWS1 in LPS tumor tissues and adjacent adipose tissues. **D** Heatmap of the correlation coefficients between the indicated components of the AKT/IWS1 phosphorylation pathway. **E** The relative stoichiometry of IWS1 in the same LPS tumor correlates with phospho-Akt (Thr 308) expression. **F** The relative stoichiometry of IWS1 in the same LPS tumor correlates with phospho-AKT (Ser 473) expression. **G** The relative stoichiometry of IWS1 phosphorylation in the same LPS tumor correlates with phospho-Akt (Thr 308) expression. **H** Immunohistochemical staining of phospho-AKT (Thr 308), phospho-AKT (Ser 473) and IWS1 in WDLPS and DDLPS tissues.

To address the clinical relevance of the AKT/p-IWS1 pathway in patients with LPS, the expression of AKT1, AKT2, AKT3, phospho-AKT (Thr 308), phospho-AKT (Ser 473), IWS1 and phospho-IWS1 (Ser 720) in 33 retroperitoneal LPS tumor specimens and 20 adjacent normal adipose tissue specimens was examined by Western blotting (Fig. 1A and Supplementary Fig. 1A). The available clinicopathologic characteristics of patients (*n* = 33) are presented in Supplementary Table 1. Increased expression of AKT1, AKT2, AKT3, IWS1 and phospho-IWS1 was observed in most LPS specimens compared to adjacent adipose tissue (Fig. 1B, C and Supplementary Fig. 1B, C). Importantly, phospho-AKT (Thr 308 and Ser 473) was higher in most tumors, even in those in which AKT1, AKT2, and AKT3 were not significantly elevated. By plotting the relative stoichiometry and comparing correlation coefficients of IWS1 and phosphorylated IWS1 with AKT, we found there were significant correlations between IWS1 and phospho-AKT (Thr 308 or Ser 473), as well as phospho-IWS1 and phospho-AKT (Thr 308) (*p* < 0.0001, *p* = 0.0416 and *p* = 0.0294 respectively, by Spearman’s rank order correlation) (Fig. 1D–G). However, there was no difference in the expression of AKT3 when comparing LPS and adjacent adipose tissue (Supplementary Fig. 1D). There were no differences in the protein expression of AKT1, AKT2, AKT3, IWS1, phospho-IWS1, phospho-AKT (Thr 308) and phospho-AKT when LPS tumors were stratified by state of differentiation (well-differentiated (WD) versus dedifferentiated (DD), Supplementary Fig. 1B, C, E). Since LPS can contain both WD and DD components, the expression of IWS1 and AKT in LPS was further explored by immunohistochemistry. While the expression of IWS1 and phospho-AKT (Thr 308 and Ser 473) were not different in DDLPS and WDLPS when examined by Western blotting, significant differences in the expression of these molecules was detected when specific DDLPS and WDLPS portions of resected tumors were examined by immunohistochemistry (*x*^2^ = 9.44, *p* = 0.002; *x*^2^ = 9.44, *p* = 0.002 and *x*^2^ = 12.86, *p* < 0.001 respectively, Fig. 1H).

**Fig. 2:**
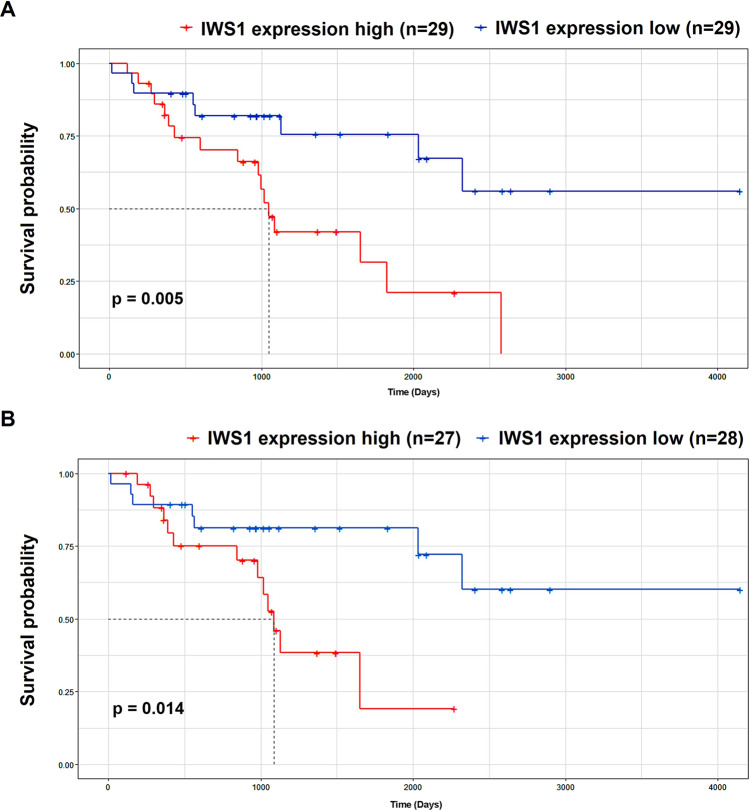
Role of IWS1 on the overall survival analysis of patients with DDLPS. **A** Kaplan–Meier overall survival analysis based on RNA-seq data of patients with DDLPS (*n* = 58) from the TCGA showed a significant reduction in overall survival (OS) and in (**B**) disease-specific survival (DSS) in patients with a higher IWS1 expression (higher than the median expression of IWS1 calculated across all samples) compared to those with a lower IWS1 expression (lower than the median, *p* = 0.014, and *p* = 0.005). **p < 0.01, ***p < 0.001 (log rank *p* test).

When we examined the oncologic outcomes of these patients, we found that an increase in phosphorylated IWS1 was associated with an increase in the number of LPS recurrences (standard error 0.112, 95% confidence interval 0.059–0.499, *p* = 0.013) and a shorter disease-free survival (DFS, HR = 3.652 *p* = 0.012), but not overall survival (Supplementary Tables 2 and 3). To further investigate this association, we carried out survival analyses based on RNA-seq data of patients with DDLPS (*n* = 58) available from TCGA. Our analysis showed a significant reduction in overall survival (OS) and disease-specific survival (DSS) in DDLPS patients with a higher IWS1 expression compared to those with a lower IWS1 expression (OS *p* value = 0.005; DSS *p* value = 0.014). The Kaplan–Meier survival curves are shown in Fig. 2A, B. Importantly, the impact of IWS1 expression of disease-free survival was not able to be determined since recurrence data is not currently available. In addition, RNA-Seq data from WDLPS samples are not available in TCGA so a comparison of patients with DDLPS was not possible. Nonetheless, our findings suggest that the AKT/p-IWS1 axis is active in LPS, and that increased expression of IWS1 is associated with a worse prognosis in patients.

### IWS1 phosphorylation at Ser720/Thr721 promotes liposarcoma colony formation

**Fig. 3:**
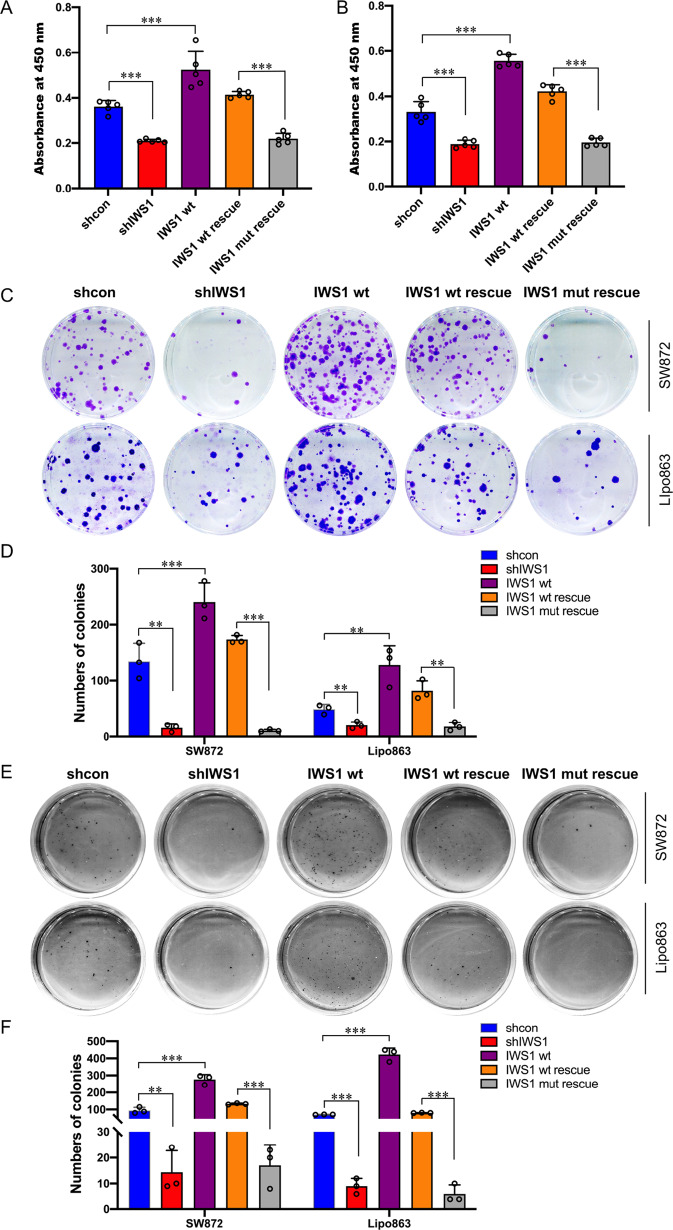
IWS1 phosphorylation at Ser720/Thr721 promote LPS colony forming capacities. **A** CCK8 assay detected the cell viability of SW872 cells with shcontrol, shIWS1, IWS1 wt, shIWS1/wt rescue or shIWS1/mut rescue transduction. **B** CCK8 assay detected the cell viability of Lipo863 cells transduced with shcontrol, shIWS1, IWS1 WT, shIWS1/WT-R or shIWS1/MT-R. **C** Plate colony forming assay detected the colony forming capacities of SW872 and Lipo863 cells transduced with shcontrol, shIWS1, IWS1 WT, shIWS1/WT-R or shIWS1/MT-R. **D** Plate colony forming analysis of the colony forming capacities in SW872 and Lipo863 cells. **E** Soft agar colony forming assay detected the colony forming capacities of SW872 and Lipo863 cells transduced with shcontrol, shIWS1, IWS1 WT, shIWS1/WT-R or shIWS1/MT-R. **F** Soft agar colony forming analysis of the colony forming capacities in SW872 and Lipo863 cells. **p < 0.01, ***p < 0.001.

To further characterize the effect of IWS1 phosphorylation by AKT on LPS tumorigenesis, we determined the rate of colony forming capacity of LPS cells stably transduced with shControl, shIWS1, and IWS1 WT constructs. The same was done in shIWS1-transduced cells rescued with the Flag-tagged wild-type IWS1 (shIWS1/WT-R) or rescued with the AKT-phosphorylation site mutant of IWS1 (Ser720A/Thr721A, shIWS1/MT-R). Notably, the ectopic Flag-tagged IWS1 clones were engineered to be shRNA-resistant [27]. Using these cells, we employed the CCK8-plate colony forming assay and soft-agar colony forming assay, growing cells in complete media under normal conditions. The knockdown efficiency and ectopic Flag-IWS1 expression were confirmed with qRT-PCR and Western blotting (Supplementary Fig. 2A–F). The loss of IWS1 expression (shIWS1) and/or phosphorylation (shIWS1/MT-R) significantly impaired plating efficiency and anchorage independent growth of Lipo863 (a dedifferentiated human LPS cell line). To determine if the observed phenotype was specific to LPS, a second experiment was performed using the undifferentiated LPS cell line, SW872 (Fig. 3A–F). In contrast to cells where expression of IWS1 was abrogated using shRNA, the plating efficiency and anchorage independent growth were noticeably enhanced by the increased expression of the wild-type IWS1 (IWS1 WT). Importantly, the impairment of cell plating efficiency and colony formation in shIWS1 LPS cells could be rescued by wild-type IWS1 (shIWS1/WT-R) but not by the mutant rescue (shIWS1/MT-R, Fig. 3A–F). These data indicate that IWS1 phosphorylation by AKT at Ser720/Thr721 promotes plating efficiency and colony formation of human LPS cell lines.

Flow cytometry using Annexin V was performed to determine if a reduction in IWS1 expression induced apoptosis and thereby reduced plating efficiency. However, there was no significant difference in shIWS1-transduced cells compared to shControl transduced cells. These findings were confirmed by performing western blot to detect the levels of cleaved caspase-3 (1:100 dilution; Cell Signaling Technology, Cat No. 9661S), protein on LPS cell lines upon IWS1 knockdown and observed no-significant difference in the expression levels of cleaved caspase-3 on shIWS1 compared to shControl cells (data not shown). To investigate how IWS1 may regulate cell growth, we used flow cytometry to characterize the impact of IWS1 expression on cell cycle progression. In three different human LPS cell lines, Lipo863, Lipo815, and Lipo224 (Supplementary Fig. 3A), there was a greater proportion of cells in the S phase of the cell cycle in shIWS1 transduced cells compared to shControl cells. Noteworthy, cells knocked-down for IWS1 (Lipo863, Lipo815, and Lipo224) had lower expression of Cyclin-dependent kinase 2 (CDK2) and cyclin A2 (CCNA2) compared to the shControl cells (Supplementary Fig. 3B). While the relationship between expression of IWS1, CDK2 and CCNA2 is unknown, we hypothesize that the impairment of cell growth and colony formation observed in shIWS1 cells is due to the downregulation in the expression levels of these important regulators of the cell cycle.

### IWS1 phosphorylation at Ser720/Thr721 promotes liposarcoma cell migration and invasion

**Fig. 4:**
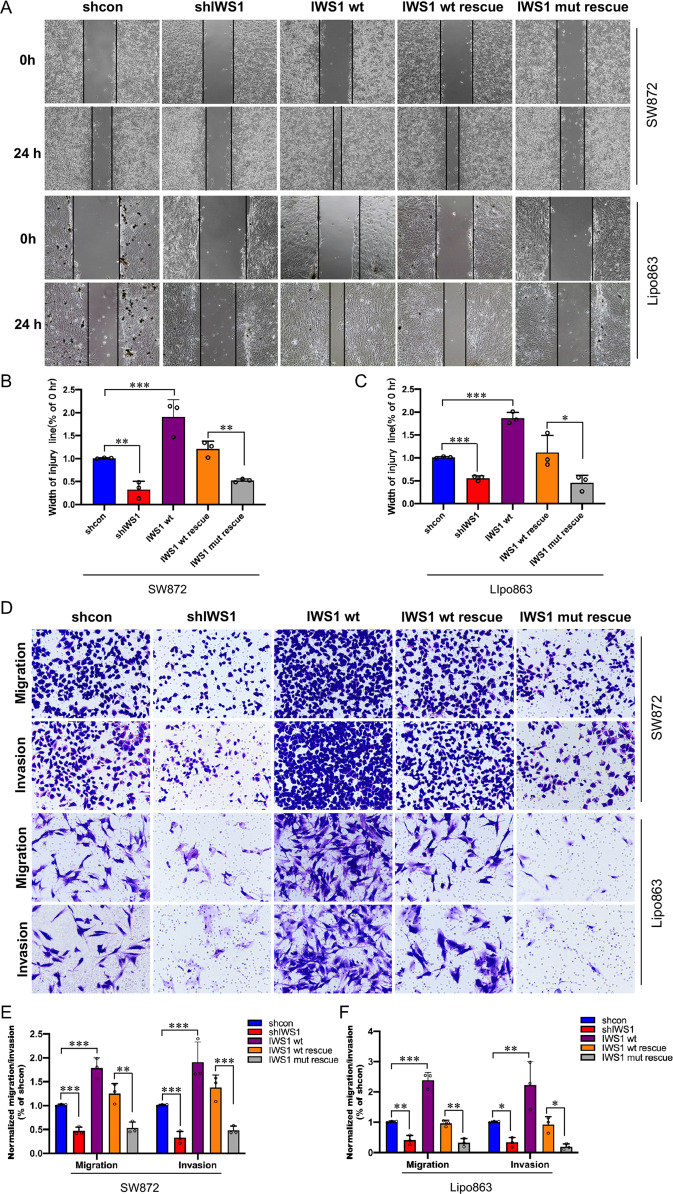
IWS1 phosphorylation at Ser720/Thr721 promote LPS cell migration and invasion. **A** Wound healing assay was conducted to detect the migratory ability of SW872 and Lipo863 cells transduced with shcontrol, shIWS1, IWS1 WT, shIWS1/WT-R or shIWS1/MT-R. **B** Wound healing analysis of the migratory ability in SW872 cells. **C** Wound healing analysis of the migratory ability in Lipo863 cells. **D** Transwell assays were performed to evaluate the migratory and invasive capacities of SW872 and Lipo863 cells transduced with shcontrol, shIWS1, IWS1 WT, shIWS1/WT-R or shIWS1/MT-R. **E** Transwell assays analysis of the migratory and invasive ability in SW872 cells. **F** Transwell assays analysis of the migratory and invasive ability in Lipo863 cells. **p < 0.05, **p < 0.01, ***p < 0.001.

To determine whether IWS1 phosphorylation by AKT impacts the migration and invasion of LPS cells, we performed wound healing and transwell assays in SW872 and Lipo863 cells following lentiviral transduction with shControl, shIWS1, IWS1 WT, shIWS1/WT-R or shIWS1/MT-R. In addition to inhibiting the plating efficiency and anchorage-independent cell growth of LPS cells, the loss of IWS1 phosphorylation also reduced cell migration and invasion. Importantly, both cell migration and invasiveness were rescued by the wild-type IWS1 (IWS1/WT-R) but not by the phosphorylation site mutant (shIWS1/MT-R) in Lipo863 cells (Fig. 4A–F). To determine if the observed phenotype was specific to LPS, a second experiment was performed using the undifferentiated sarcoma cell line, SW872. We also conducted migration and invasion assays on other LPS cell lines that overexpress mouse double minute 2 (MDM2), such as Lipo224 and Lipo815 cells, transduced with shControl, shIWS1, and IWS1 WT. There was a significant decrease in cell migration and invasion of both Lipo224 and Lipo815 (Supplementary Fig. 4A–C) cells that were transduced with shIWS1 compared to the shControl cells. In contrast, the previously mentioned cell lines transduced with IWS1 WT exhibited an opposite phenotype by showing enhanced migration and invasiveness. Taken together, our findings clearly demonstrate the impact of IWS1 phosphorylation at Ser720/Thr721 by AKT in cell migration and invasion of LPS.

### IWS1 phosphorylation Ser720/Thr721 promotes tumor growth and metastasis of liposarcoma in vivo

**Fig. 5:**
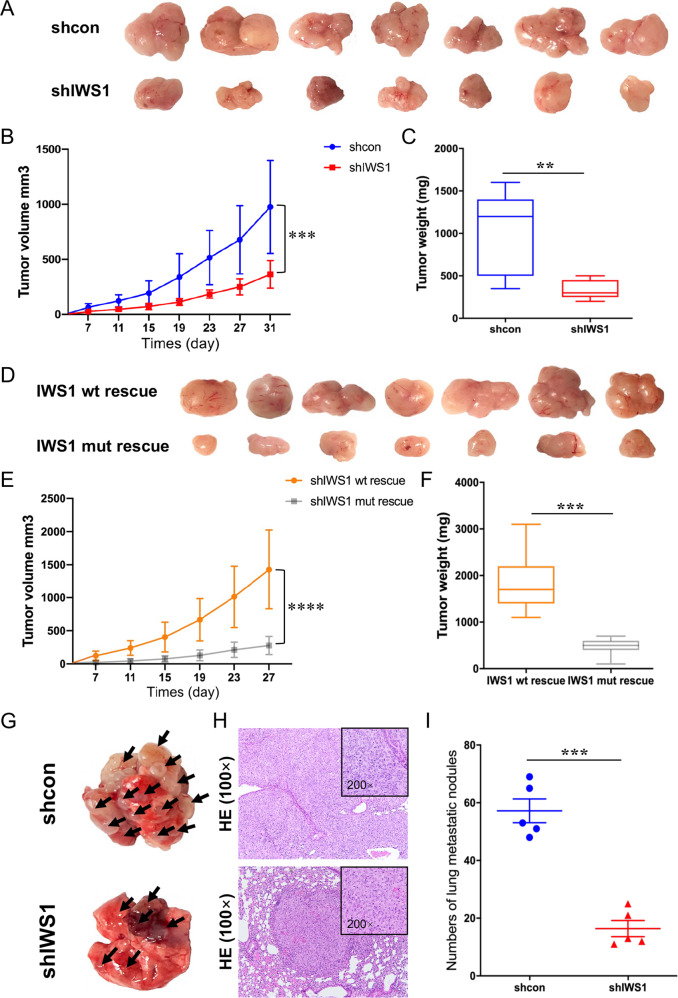
IWS1 phosphorylation at Ser720/Thr721 promotes tumor growth in vivo, while IWS1 knockdown inhibited tumor growth and metastasis in vivo. **A** Xenograft LPS model of nude mice inoculated with shcontrol and shIWS1 SW872 cells. **B** Tumor growth curves of nude mice inoculated with shcontrol and shIWS1 SW872 cells. **C** Tumor weights of nude mice inoculated with shcontrol and shIWS1 SW872 cells. **D** Xenograft LPS model of nude mice inoculated with shIWS1/WT-R and shIWS1/MT-R SW872 cells. **E** Tumor growth curves of nude mice inoculated with shIWS1/WT-R and shIWS1/MT-R SW872 cells. **F** Tumor weights of nude mice inoculated with shIWS1/WT-R and shIWS1/MT-R SW872 cells. **G** LPS metastasis model of nude mice with tail vein injection of shcontrol and shIWS1 SW872 cells. **H** H&E staining of the lung metastatic nodules in LPS metastasis model of nude mice. **I** Lung metastatic nodules analysis in LPS metastasis model of nude mice.

To address the role of IWS1 phosphorylation in sarcoma tumor growth in vivo, we injected 5 × 10^6^ shControl, shIWS1, shIWS1/WT-R or shIWS1/MT-R SW872 cells in immunocompromised NSG mice. Cells were injected either subcutaneously (sc) or intravenously (IV-tail vein). The animals were monitored for tumor development and lung metastasis for 4 and 6 weeks, respectively. Consistent with our in vitro data, the results revealed that the knockdown of IWS1 resulted in reduced tumor growth (tumor volume and weight) and expression of Ki 67, with a parallel reduction in the number of metastatic lung nodules (Fig. 5A–C, G–I and Supplementary Fig. 5A, B). Importantly, the observed reduction in tumor growth, expression of Ki 67, and lung metastases were rescued in the shIWS1/WT-R tumor bearing animals, but not in the shIWS1/MT-R (Fig. 5D–F and Supplementary Fig. 5A, B). The knockdown of IWS1 and its rescue by the wild-type and mutant IWS1 were validated by western blotting of human cell lysates (Supplementary Fig. 5C).

Western blots of the same cell lysates also showed that the knockdown of IWS1 resulted in the downregulation of the EMT markers E-Cadherin, Occludin, N-Cadherin, and Vimentin, the EMT-promoting factors SNAIL and SLUG, the stemness factors FLF3, Nanog, OCT4 and Sox2. Moreover, whereas the wild-type IWS1 rescued the expression of these proteins, the mutant IWS1 failed to do so (Supplementary Fig. 5C). Downregulation of Vimentin, along with the expression of cytokeratin, which is also downregulated in shIWS1 and shIWS1/MT-R xenografts, were also examined by immunohistochemistry (Fig. 5D).

### IWS1 phosphorylation at Ser720/Thr721 promotes a mesenchymal-to-epithelial plasticity and cancer stem cell-renewal program in human liposarcoma cell lines

The PIK3/AKT pathway has been associated with EMT in several human cancers, through direct phosphorylation, or indirectly, through transcriptional regulation of EMT effectors [40–42]. Recent evidence suggests that EMT is reversible, and a strong link exists between EMT/MET and CSC formation [12, 15]. EMT/MET programs confer CSC-like characteristics to cells within the tumor and are important drivers of tumoral heterogeneity and resistance to currently available therapies [17, 18, 43]. As such, we examined whether the loss of IWS1 or phosphorylated IWS1 alters the expression of EMT markers and transcription factors association with EMT.

**Fig. 6:**
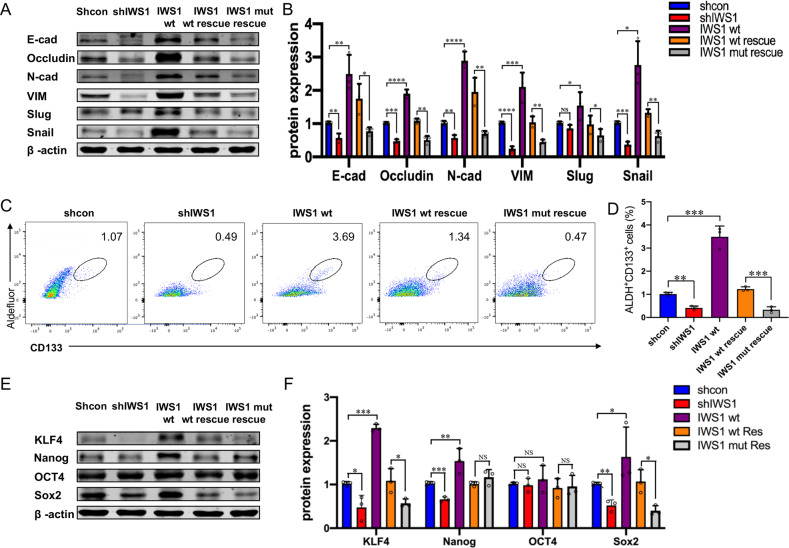
IWS1 phosphorylation at Ser720/Thr721 represses MET and promotes cancer stem cell-like properties in LPS cells. **A** Western blot of the MET-associated markers E-cad, Occludin, N-cad, VIM, Snail and Slug in SW872 cells transduced with shcontrol, shIWS1, IWS1 WT, shIWS1/WT-R or shIWS1/MT-R. **B** Graphical representation of Western blot showing the expression of MET-associated marker E-cad, Occludin, N-cad, VIM, Snail and Slug in SW872 cells. **C** Flow cytometry was performed to evaluate the ALDH^+^/CD133^+^ subpopulations in SW872 cells transduced with shcontrol, shIWS1, IWS1 WT, shIWS1/WT-R or shIWS1/MT-R. **D** Flow cytometry analysis of ALDH^+^/CD133^+^ subpopulations in SW872 cells. **E** Western blot of CSC marker KLF4, Nanog, OCT4 and Sox2 in SW872 cells. **F** Western blot analysis the expression of CSC marker KLF4, Nanog, OCT4 and Sox2 in SW872 cells. **p < 0.01, ***p < 0.001.

Occludin and E-cadherin are markers of epithelial cells and are downregulated in EMT. N-Cadherin and Vimentin are markers of mesenchymal cells and are induced in cells undergoing EMT. The Western blot in Fig. 6A, B shows that knockdown of IWS1 downregulates both E-Cadherin and Occludin, as well as N-Cadherin and Vimentin. This suggests IWS1 inhibits both the MET and EMT program in the undifferentiated LPS cell line SW872, thereby maintaining plasticity and tumoral heterogeneity. In further support, the overexpression of IWS1 gave rise to the opposite phenotype. Importantly, the phosphorylation site IWS1 mutant failed to rescue the phenotype of the IWS1 knockdown. This suggests that the downregulation of these markers is IWS1-phosphorylation dependent and suggest that IWS1 phosphorylation may promote both the MET and the EMT transition resulting in a population of “metastable” cells. To this end, the results in Fig. 6A, B also shows that the expression of the EMT-promoting transcription factors Snail and Slug follow the same pattern as the expression of the MET and EMT markers upon the knockdown or overexpression of IWS1 in these same cell lines.

Cells undergoing EMT tend to exhibit stem cell properties [16]. This, combined with the observation that AKT signaling has been linked to maintenance of stem cells in LPS and that IWS1 has been shown to regulate the expression of Nanog, a stemness promoting factor, raised the question of whether IWS1 regulates the stemness of LPS cell lines [22, 28]. To address these questions, we used flow cytometry to determine whether the knockdown of IWS1 and its rescue by the phosphorylation site IWS1 mutant affected the relative number of cells expressing the LPS stem cell markers ALDH and CD133 [9, 10]. Our results showed that that a small percentage of shControl SW872 cells (0.92%–1.15%) display high CD133 expression and ALDH activity (Fig. 6C, D). Importantly, the reduced percentage of stem cells was rescued in shIWS1/WT-R, but not in shIWS1/MT-R cells (Fig. 6C, D). Next, Western blotting was performed to determine whether the knockdown of IWS1 and its rescue by the phosphorylation site IWS1 mutant affected the stemness promoting transcription factors KLF4, Nanog, OCT4, and Sox2 in SW872 cells (Fig. 6E, F). These data indicate that the IWS1 phosphorylation at Ser720/Thr721 by AKT kinase promotes EMT/MET plasticity and cancer stem cell-like properties in LPS cells.

## Discussion

In this study we demonstrate that phosphorylation of the transcription elongation factor, IWS1, by AKT is an important cellular event that maintains CSCs and mesenchymal-epithelial plasticity in human LPS. Specifically, phosphorylated IWS1 maintains levels of Nanog, the presence of CD133+ CSCs and EMT status in both cell and xenograft models of LPS and is required for anchorage-independent growth, migration, invasion, and tumor metastases. In patients with LPS, IWS1 expression is associated with a reduction in overall survival, and the presence of phosphorylated IWS1 is associated with an increase in the number of tumor recurrences and a shorter time to relapse.

Mechanistically, phosphorylation of IWS1 by AKT3 and AKT1 at Ser720/Thr721 recruits the Histone H3K36 trimethyl-transferase, SETD2, to the RNA Poll II complex. Together these proteins create a docking site for MRG15 and PTB that is required for transcription elongation [27]. This study confirms a similar role of PI3K/AKT signaling and IWS1 phosphorylation in the cell biology and tumorigenesis of human LPS.

Dysregulation of PI3K/AKT signaling has long been implicated in the aggressive clinical behavior of LPS and other types of soft tissue sarcoma [20, 21]. We found that phosphorylation of the transcription elongation factor, IWS1, by AKT is an important cellular event required for anchorage-independent growth, migration, invasion, and in vivo tumor metastases in preclinical models of human LPS. Moreover, the results are consistent in undifferentiated cell lines of LPS and in those with MDM2 amplification, suggesting that the impact of IWS1 on LPS phenotype is independent of MDM2. In addition, a retrospective analysis of patients treated at our center found that increased levels of phosphorylated IWS1 in resected tumors was associated with an increase in the number of tumor recurrences and a shorter disease-free interval in patients with retroperitoneal LPS. This finding is consistent with previous studies on AKT in LPS but is the first to implicate the transcription elongation factor IWS1 and its phosphorylation by AKT as a critical event in maintaining an aggressive phenotype.

LPS originate from the mesenchymal stem cell population, and it is well known that these CSCs are maintained as tumors progress. In fact, grade 4 dedifferentiated LPS express stem cell markers [44]. It is believed that these CSCs confer resistance to chemo and radiotherapy that is so characteristic of LPS [23]. In our study, we found that the phosphorylation of IWS1 maintains Nanog expression and CD133+ CSC phenotypes in human LPS cell lines and xenograft models. Nanog is an important molecule for tumor growth and progression in sarcomas and confers resistance to Doxorubicin [22, 45]. Consistently, we observed that IWS1 phosphorylation promotes tumor growth and lung metastasis in vivo. These results are consistent with previous separate reports in the literature, whereby AKT kinase maintains Nanog expression and CSCs in sarcoma, and where phosphorylation of IWS1 is necessary for Nanog expression in murine embryonic development [22, 28]. The results of this study suggest that IWS1 phosphorylation by AKT is an important event for maintaining Nanog expression in human LPS and based on recent findings where inhibition of CSCs by targeting AKT reduced chemo and radio resistance, may hold potential as a novel therapeutic target. Targeting IWS1 specifically may circumvent the toxicity of currently available AKT inhibitors, while reducing resistance to chemo and/or radiotherapy by reversing CSC phenotypes in patients with LPS and other forms of sarcoma [46–48]. On the other hand, since IWS1 is ubiquitously expressed, its inhibition may lead to significant toxicity [39].

Increased cell migration and invasiveness in epithelial cell-derived tumors is commonly the result of EMT. During EMT cells lose the epithelial phenotype and become more migratory and invasive by acquiring a mesenchymal phenotype [16]. Recent studies have shown that this is a multifaceted process rather than a single, linear event and is implicated in cancer progression, metastasis, and drug resistance [15, 16]. Importantly, this process is reversible and tumor cells can undergo MET whereby they acquire a more sessile and proliferative phenotype like that of epithelial cells [12]. Sarcomas are characterized by their mesenchymal features. As such, EMT in sarcomas may seem like a paradox [12]. However, many sarcomas are heterogeneous, with many of their cells having an intermediate phenotype that express both epithelial and mesenchymal markers [13, 49–51]. The presence of cells exhibiting this intermediate “meta stable” phenotype is considered a feature of tumors with more aggressive tumor biology because these cells can undergo MET and give rise to highly proliferative cells in the primary tumor, or EMT, giving rise to migratory, invasive, and metastatic cells [12].

In the present study, we found that the knockdown of IWS1 downregulates both E-Cadherin and Occludin, as well as N-Cadherin and Vimentin, suggesting that it inhibits both the MET and the EMT program in the undifferentiated LPS cell line SW872. More so, overexpression of IWS1 gave rise to the opposite phenotype, supporting the conclusion that IWS1 may promote a “metastable” population of cells. Importantly, the phosphorylation site IWS1 mutant failed to rescue the phenotype of the IWS1 knockdown and suggests that the downregulation of these markers is IWS1-phosphorylation dependent. In further support, the expression of the EMT-promoting transcription factors Snail and Slug, both of which are canonically downstream of PI3K/AKT, followed the same pattern of expression as the MET/EMT markers upon knockdown or overexpression of IWS1 in these same cell lines. Lastly, phosphorylation of IWS1 was required for anchorage-independent growth, migration and invasion, and tumor metastases. These findings are the first to implicate AKT-dependent phosphorylation of the transcription elongation factor IWS1 in the maintenance of this “metastable” phenotype and increased tumorigenesis in cancer.

While the exact mechanism of how IWS1 exerts its effects on LPS cells is unknown, our results also suggests that phosphorylation of IWS1 by AKT may play a role in cell cycle progression. AKT is an important regulator of the cell cycle by maintaining protein stability of during multiple cell cycle checkpoints [52]. We found that knockdown of the AKT-target, IWS1, resulted in a downregulation of CDK2 and cyclin A2 expression and lead to a greater proportion of cells in the S phase. By altering cell cycle dynamics, IWS1 may impact cell viability, colony formation, cell migration and invasion.

In conclusion, the phosphorylation of IWS1 by AKT is an important cellular event that maintains tumoral heterogeneity and the oncogenic potential of human LPS by maintaining CSCs and EMT/MET plasticity. These findings support the use of the AKT/IWS1 axis as a novel prognostic factor and potential therapeutic target in patients with advanced LPS.

## Materials and methods

### Clinical specimens

A total of 33 primary or recurrent LPS tumor tissue samples and 20 matched adjacent adipose tissue samples, were acquired from patients who underwent surgical resections from 1992 to 2019 at The Ohio State University Wexner Medical Center and included 18 of well-differentiated retroperitoneal LPS and 15 of dedifferentiated retroperitoneal LPS. Each case was validated and classified by an experienced bone and soft tissue pathologist (OHI) based on the most recent World Health Organization (WHO) classification of tumors. This study was approved by the Institutional Review Board of The Ohio State University Wexner Medical Center (IRB approval No: 2017C0139) and written informed consent was provided by all participants. All specimens were frozen and stored at −80 °C immediately.

### Immunohistochemistry

Immunohistochemistry was carried out on 4 μm pathological paraffin sections and stained with a DAB Peroxidase (HRP) Substrate Kit (Vector Labs, Cat No. SK-4100) for the detection of p-AKT (T308) (1:100 dilution; Cell Signaling Technology, Cat No. 13038), p-AKT (S473) (1:100 dilution; Cell Signaling Technology, Cat No. 4060), IWS1 (1:100 dilution; Cell Signaling Technology, Cat No. 5681), Vimentin (1:100 dilution; Cell Signaling Technology, Cat No. 5741S), pan-Cytokeratin (1:100 dilution; Abcam, Cat No. ab80826) and Ki-67 (1:100 dilution; Cell Signaling Technology, Cat No. 9027S) according to the manufacturer’s protocols. The staining intensity was graded independently by two experienced pathologists.

### Cell culture

The human LPS cell line SW872 was obtained from ATCC, and the Lipo863, Lipo224, Lipo815 cell lines were established in our laboratory as previously reported [53]. Frozen stocks are re-analyzed every 6 months for any changes in cell biology. All cell lines utilized in the study have been tested for mycoplasma and undergo testing every month utilizing both biochemical chemiluminescent assay and PCR based assay systems. All cells were cultured in Dulbecco’s modified Eagle’s medium (DMEM; Corning, Cat No. 10-013-CV) supplemented with 10% fetal bovine serum (FBS; Corning, Cat No. 35-011-CV) and 1% penicillin/streptomycin (Gibco, Cat No. 15140-122) at 37 °C in a humidified atmosphere containing 5% CO_2_. Cell lines were also periodically checked for mycoplasma, using the PCR mycoplasma detection kit (ABM, Cat No. G238). All experiments were carried out in mycoplasma-free cultures.

### Transfections and infections

Packaging plasmid psPAX2 (Addgene #12260), enveloping plasmid pMΔ2.G (Addgene #12259), and three lentiviral vectors including shRNA against human IWS1, pLx304 Flag-IWS1, pLx304 Flag-IWS1 S720A/T721A mutant (shRNA-resistant clones) were used [27]. Lentiviruses were packaged in HEK-293T cells by transient transfection of lentiviral constructs and packaging constructs using 2x HEPES Buffered Saline (Sigma Cat. No 51558) and calcium-phosphate precipitation method. Lentiviral supernatants were collected at 48 h post transfection and filtered through a 0.45 μm filter. LPS cells were infected with viral particles in the presence of 8 μg/ml polybrene (Sigma, Cat No. 107689) for 8 h and selected for stable infection with 2 μg/ml puromycin (MilliporeSigma, Cat No 54-041-1.) or 5 μg/ml blasticidin (MilliporeSigma, Cat No. 20-335-1). Cells infected with multiple constructs were selected for infection with the first construct, prior to the next infection. The pLKO.1 TRC control vector was utilized as a non-hairpin control (Addgene #10879) [27].

### Cell viability assay

LPS cells were seeded and cultured in 96-well plates at a density of 2 × 10^4^/well for 24 h. Cell viability was assessed by the Cell counting kit-8 (CCK8) assay kit (Dojindo Molecular Technologies, Cat No. CK0401) with the absorbance measured at 450 nm using a multi-mode microplate reader.

### Colony formation assay

For the plate colony formation assay, stable transfected SW872 cells (0.5 × 10^3^) or Lipo863 cells (1 × 10^3^) were seeded in 60-mm culture dishes and cultured in DMEM medium containing 10% FBS. Following incubation at 37 °C for 2 weeks, the colonies were fixed with methanol for 20 min and stained with 0.5% crystal violet for 10 min. For Soft Agar colony formation assay, stable infected SW872 cells or Lipo863 cells were mixed into DMEM medium containing 10% FBS and 0.35% Noble agar (Difco™ Agar Noble, BD Biosciences, Cat. No DF0142-15-2) and seeded in 60-mm culture dishes pre-coated with 0.6% noble agarose in DMEM medium containing 10% FBS. The cells in the agar dish were incubated at 37 °C and then stained with MTT for 20 min. The images of the colonies were obtained by a scanner (Epson America Inc., Long Beach, CA) and the number of colonies was quantified with ImageJ software.

### Wound healing assay

Stable transfected LPS cells were seeded and inoculated in 6-well plates until the cell reached more than 95% confluence. The monolayer was scratched with a sterile 100 μl pipette tip and the wound closure was observed under an inverted microscope at 0 and 48 h after wounding.

### Transwell assay

Stable transfected LPS cells (2 × 10^4^) were placed into the upper chamber (8.0-μm pore size; Corning Inc., Cat No. 3422) uncoated or pre-coated with Matrigel (Corning Inc., Cat. No 354234) to identify the cell migration and invasion. Following incubation for 24 h, the number of cells penetrating to the underside of the membrane were stained with crystal violet and visualized with an inverted microscope.

### Quantitative polymerase chain reaction (RT-qPCR)

Total RNA was extracted from LPS cells using a NucleoSpin RNA kit (Macherey-Nagel, Duren, Cat. No 740955.5) and synthesized into cDNA using 5 × Prime Script RT Master Mix (Takara Bio, Inc., Cat. No RR036A) under the following conditions: 37 °C for 15 min, 85 °C for 5 s and 4 °C. RT-qPCR was conducted on the StepOne Real-Time PCR System (Applied Biosystems, Cat. No A25742) with specific primers and SYBR green mix under the following conditions: 15 min at 95 °C, followed by 40 cycles of 5 sec at 95 °C, 30 s at 60 °C and 60 s at 72 °C. Relative expression was evaluated using the 2^−ΔΔCt^ method with GAPDH as an endogenous control. The primers were listed as follows: IWS1 forward, 5ʹ-GAACAGCACTGGTGGTCAGACACC-3ʹ and reverse, 5ʹ-TGGCACCAGTGTCCGGATTA-3ʹ; CDK2 forward, 5ʹ-ATGGATGCCTCTGCTCTCACTG-3ʹ and reverse, 5ʹ-CCCGATGAGAATGGCAGAAAGC-3ʹ; CCNA2 (Cyclin A2) forward, 5ʹ-CTCTACACAGTCACGGGACAAAG-3ʹ and reverse, 5ʹ- CTGTGGTGCTTTGAGGTAGGTC-3ʹ; GAPDH forward, 5ʹ-TTCGACAGTCAGCCGCATCTTCTT-3ʹ and reverse, 5ʹ-CAGGCGCCCAATACGACCAAATC-3ʹ.

### Western blot

Total protein of LPS tissue and cells were lysed by T-PER Tissue Protein Extraction Reagent (Thermo Scientific, Cat. No 78510) or CelLytic TM M cell lysis reagent (Sigma-Aldrich, Cat. No C2978) supplemented with Halt™ Protease and Phosphatase Inhibitor Cocktail (Thermo Scientific, Cat. No 78438 and 78428), separately. Equal amounts of proteins were subjected to SDS-PAGE gel and transferred onto nitrocellulose membrane. After blocking with 5% BSA, the membranes were incubated with primary antibodies specific for AKT1 (2938S), AKT2 (3063S), AKT3 (14982S), p-AKT (T308) (13038S), p-AKT (S473) (4060S), pan-AKT (4685S), IWS1 (5681S), E-cadherin (3195S), Occludin (91131S), N-cadherin (13116S), Vimentin (5741S), Slug (9585S), Snail (3879S), KLF4 (12173S), Sox2 (2748S), OCT4 (2750S), Nanog (3580S), and β-actin (8457S) were purchased from Cell signaling and then incubated with IRDye 800CW-conjugated secondary antibodies (1:5000, LI-COR Biosciences, Cat. No 926-32211). Protein bands were visualized with an Odyssey Infrared Imaging System (LI-COR Biosciences). Antibody specific for p-IWS1 was kindly gifted by Dr. Philip Tsichlis [27].

### Flow cytometry analysis of cancer stem cell markers

Stable transfected LPS cells (1 × 10^6^/ml) were stained with Aldefluor™ kit (Stem Cell Technologies Inc., Cat. No 01700) for 45 min at 37 °C and then incubated with allophycocyanin (APC) conjugated anti-human CD133 antibodies (1:100, Biolegend, Cat. No 372806) for 30 min at 4 °C according to the manufacturer’s instructions. A specific ALDH inhibitor Diethylaminobenzaldehyde (DEAB) was used as a negative control (Info). Flow cytometry analysis was conducted on a LSR Fortessa Flow Cytometer (BD Biosciences, San Jose, CA) with FACS Diva Software (Version 8.0.1, BD Pharmingen) and analyzed using Flowjo software (version 10).

### In vivo tumorigenesis and metastasis assays

Male athymic nude mice (NCr-nu/nu, 4–6 weeks) were obtained from Target Validation Shared Resource at The Ohio State University and randomization was not used to determine how animals were allocated to experimental groups. For xenograft tumor model, 5 × 10^6^ shControl, shIWS1, shIWS1/WT-R or shIWS1/MT-R SW872 cells were suspended in 100 μl PBS containing 50% Matrigel and inoculated subcutaneously into the flank of nude mice. Tumor size was measured every 3 days and tumor volume was calculated based on the formula: ${V} = \frac{{{{{\mathrm{length}}}} \times {{{\mathrm{width}}}}^2}}{2}$. On day 31, xenografts were harvested, fixed in 10% formalin, and then embedded in paraffin. For the lung metastasis model, 1 × 10^6^ shControl or shIWS1 SW872 cells were injected into the nude mice through the tail vein. Six weeks after injection, lung tissues with metastatic nodules were observed in gross and confirmed by hematoxylin & eosin (H&E) staining. The animal experimental procedures were approved by the Institutional Animal Care and Use Committee of The Ohio State University under protocol number 2013R00000039 and no blinding was done during all stages of animal experiment.

### Survival analysis on TCGA DDLPS data

The raw read counts (coding and non-coding RNAs) and clinical data for the TCGA-SARC cohort were downloaded via the GDC data portal. Overall, all DDLPS samples (*N* = 58) available from the TCGA-SARC cohort were selected. Raw counts were scaled using the Read Per Million (RPM) mapped reads formula and genes with a geometric mean of RPM < 5 were removed because they were not expressed or expressed at very low levels. After the filtering step, we normalized the expressed genes with the Trimmed Mean of M values (TMM) method using the edgeR R package [54] and we selected IWS1 for carrying out OS and DSS analyses by employing a univariate Cox regression model leveraging the *survFit* function from the Survival R package [https://cran.r-project.org/web/packages/survival/index.html]. The median of IWS1 expression across all TCGA-DDLPS samples was used as threshold to define the group of patients with high and low IWS1 expression. Kaplan–Meier curves were finally generated using the *ggsurvplot* function from Survminer R package [https://cran.r-project.org/web/packages/survminer/index.html].

### Cell cycle analysis

The DNA content of Stable transfected LPS cells was determined using FxCycle™ PI/RNase Staining Solution (Invitrogen; F10797). Cells of 2 × 10^5^ per sample were fixed and stained with staining solution for 15 min for analyzing DNA content. Then, the cell samples were analyzed using LSR Fortessa Flow Cytometer (BD Biosciences, San Jose, CA) and then analyzed using a FACSDiva software (Version 6.1.3).

### Apoptosis analysis

The Stable transfected LPS cells were washed with 1X PBS and resuspended in a binding buffer at a concentration of 2 × 10^5^ cells. The eBioscience™ Annexin V-FITC Apoptosis Detection Kit (Invitrogen; BMS500FI) was purchased, and the apoptosis detection assay was performed by staining with Annexin V-FITC at room temperature for 10 min and then stained with propidium iodide. The assay was performed with LSR Fortessa Flow Cytometer (BD Biosciences, San Jose, CA), and apoptotic cells were analyzed using a FACSDiva software (Version 6.1.3).

### Statistical analysis

Statistical analysis was performed using SPSS 20.0 software (IBM Corp., Armonk, NY, USA) and GraphPad Prism 9.0 (GraphPad Inc., La Jolla, CA, USA). Two-tailed Student’s *t* test or one-way analysis of variance (ANOVA) was conducted to analyze the significance between variables. Data are presented as mean ± standard deviation (SD). Cox proportional hazard model (accounting for age, size of tumor, grade, and differentiation) was used to evaluate the effects of IWS1 expression on overall and disease-free survival. Overall survival was calculated from time of diagnosis to death and recurrent free survival was calculated from time of resection to date of first recurrence. Negative binomial regression was employed to assess the effects of IWS1 on the number of deaths. To minimize the effects of the extreme’s values of IWS1, logarithmic transformation was used in the model. *p* < 0.05 was considered as statistically significant difference. All statistical tests we used in every figure are justified as appropriate and the data meet the assumptions of the tests as normal distribution.

## Supplementary information

Supplementary file legends

Supplementary Table 1

Supplementary Table 2

Supplementary Table 3

Supplementary Figure 1

Supplementary Figure 2

Supplementary Figure 3

Supplementary Figure 4

Supplementary Figure 5

## Data Availability

All data associated with this study are present in the paper or the Supplementary Materials.
