# Supplementary material for: Phosphorylation of IWS1 by AKT maintains liposarcoma tumor heterogeneity through preservation of cancer stem cell phenotypes and mesenchymal-epithelial plasticity
Source: Oncogenesis. 2023 May 26;12(1):30. doi: 10.1038/s41389-023-00469-z (PMC10219984; doi:10.1038/s41389-023-00469-z)
Supplement: Supplementary file 1 — Supplementary file legends [file 41389_2023_469_MOESM1_ESM.docx]

**Supplementary file legends**

**Supplementary Fig. 1.** AKT/IWS1 axis is active in human liposarcoma. (A) Western blot of AKT1, AKT2, AKT3, phospho-AKT (Thr 308), phospho-AKT (Ser 473), pan-AKT, IWS1, and phospho-IWS1 in LPS tumor tissues and adjacent adipose tissues. (B) Western blot analysis of the expression of phospho-AKT (Thr 308) and phospho-AKT (Ser 473) in WDLPS and DDLPS tissues. (C) Western blot analysis of the expression of IWS1 and phospho-IWS1 in WDLPS and DDLPS tissues. (D) Western blot analysis of the expression of AKT1, AKT2, and AKT3 in LPS tumor tissues and adjacent adipose tissues. (E) Western blot analysis of the expression of AKT1, AKT2 and AKT3 in WDLPS and DDLPS tissues. *P<0.05, **P<0.01, ***P<0.001.

**Supplementary Fig. 2.** The mRNA and protein expression of IWS1 in the SW872 and Lipo863 cells after transduction. (A) qRT-PCR was conducted to detect the mRNA expression of IWS1 in SW872 cells transduced with shcontrol, shIWS1, IWS1 WT, shIWS1/WT-R or shIWS1/MT-R. (B) qRT-PCR was conducted to detect the mRNA expression of IWS1 in Lipo863 cells transduced with shcontrol, shIWS1, IWS1 WT, shIWS1/WT-R or shIWS1/MT-R. (C) Western blot of IWS1 in SW872 cells transduced with shcontrol, shIWS1, IWS1 WT, shIWS1/WT-R or shIWS1/MT-R. (D) Western blot of IWS1 in Lipo863 cells transduced with shcontrol, shIWS1, IWS1 WT, shIWS1/WT-R or shIWS1/MT-R. *P<0.05, **P<0.01, ***P<0.001. (E) Western blot analysis of the expression of IWS1 in SW872 cells. (F) Western blot analysis of the expression of IWS1 in Lipo863 cells. *P<0.05, **P<0.01, ***P<0.001.

**Supplementary Fig. 3.** IWS1 knock-down induces cell cycle arrest. (A) Flow cytometry analysis of Lipo863, Lipo815, and Lipo224 cells transduced with shcontrol and shIWS1. Graphical representation of flow cytometry analysis showing the accumulation of cells in the S phase of the cell cycle in all three cell lines that were transduced with shIWS1. *P<0.05, **P<0.01, ***P<0.001. (B) qRT-PCR was conducted to detect the mRNA expression of S phase checkpoint genes, CDK2 and CCNA2 (Cyclin A2) on Lipo863, Lipo815, and Lipo224 cells transduced with shcontrol, and shIWS1. *P<0.05, **P<0.01, ***P<0.001.

**Supplementary Fig. 4.** IWS1 promotes liposarcoma cell migration and invasion. (A) Transwell assays were performed to evaluate the migratory and invasive capacities of Lipo224 and Lipo815 cells transduced with shcontrol, shIWS1, or IWS1 WT. (B) Transwell assays analysis of the migratory and invasive ability in Lipo224 cells. (F) Transwell assays analysis of the migratory and invasive ability in Lipo815 cells. *P<0.05, **P<0.01, ***P<0.001.

**Supplementary Fig. 5.** IWS1 phosphorylation at Ser720/Thr721 promotes tumor growth in vivo. (A) Immunohistochemical staining of Ki 67 in tumor xenografts. (B) Graphical representation of Immunohistochemical staining of Ki 67 in tumor xenografts. **P<0.01, ***P<0.001. (C) Western blot of IWS1, MET-associated markers E-cad, Occludin, N-cad, VIM, Snail and Slug as well CSC marker KLF4, Nanog, OCT4 and Sox2 and expression of CSC markers KLF4, Nanog, OCT4 and Sox2 in tumor xenografts. (D) Graphical representation of Western blot analysis showing expression of the MET-associated markers E-cad, Occludin, N-cad, VIM, Snail and Slug in tumor xenografts. (E) Graphical representation of Western blot analysis showing the expression of IWS1 and the expression of CSC markers Sox2, Nanog, KLF4, and OCT4 in tumor xenografts. (F) Immunohistochemical staining of VIM and pan Cytokeratin in tumor xenografts. **P<0.01, ***P<0.001.

**Supplementary Table 1:** Clinicopathologic variables of patients with resected retroperitoneal liposarcoma.

**Supplementary Table 2:** Association of IWS1 expression with disease-free survival in patients with retroperitoneal liposarcoma.

**Supplementary Table 3:** Association of IWS1 expression with overall survival in patients with retroperitoneal liposarcoma.
