# Supplementary material for: Phosphorylation of IWS1 by AKT maintains liposarcoma tumor heterogeneity through preservation of cancer stem cell phenotypes and mesenchymal-epithelial plasticity
Source: Oncogenesis. 2023 May 26;12(1):30. doi: 10.1038/s41389-023-00469-z (PMC10219984; doi:10.1038/s41389-023-00469-z)
Supplement: Supplementary file 2 — Supplementary Table 1 [file 41389_2023_469_MOESM2_ESM.docx]

| **Patient** | **Age** | **Sex** | **ASA Class** | **Primary Tumor/Local Recurrence** | **Radiation Therapy** | **Systemic Therapy** | **Tumor Size (cm)** | **FNCLCC**  **Grade** | **Number of Recurrences** | **Recurrence Free Survival (months)** | **Overall Survival**  **(months)** |
| --- | --- | --- | --- | --- | --- | --- | --- | --- | --- | --- | --- |
| 1 | 61 | F | 3 | Primary | No | - | 30 | 1 | 0 | - | 23 |
| 2 | 72 | M | 2 | Recurrence | No | Yes | 9 | 3 | 1 | 25 | 25 |
| 3 | 59 | F | 3 | Recurrence | No | No | 7 | 2 | 1 | 5 | 31 |
| 4 | 45 | F | 2 | Primary | No | No | 12.3 | 1 | 0 | - | 19 |
| 5 | 78 | F | 3 | Recurrence | No | Yes | 29 | 2 | 1 | 20 | 44 |
| 6 | 70 | M | 3 | Primary | No | No | 10 | 1 | 0 | - | 13 |
| 7 | 69 | M | 4 | Primary | No | Yes | 35.5 | 2 | 2 | 13 | 43 |
| 8 | 80 | M | 4 | Primary | Yes | Yes | 22 | 3 | 2 | 30 | 48 |
| 9 | 77 | F | - | Recurrence | No | Yes | 23 | 3 | 1 | 7 | 50 |
| 10 | 64 | F | 3 | Recurrence | No | Yes | 27 | - | 3 | 18 | 54 |
| 11 | 59 | M | 3 | Recurrence | No | No | 7 | 1 | 2 | 6 | 56 |
| 12 | 76 | M | 3 | Primary | No | No | 24.5 | 1 | 0 | - | 54 |
| 13 | 81 | F | 3 | Primary | Yes | No | 10.0 | 3 | 0 | - | 20 |
| 14 | 59 | M | 3 | Primary | No | No | 20.2 | 3 | 3 | 40 | 76 |
| 15 | 75 | F | - | Recurrence | No | Yes | 15 | - | 3 | 13 | 82 |
| 16 | 75 | F | - | Recurrence | Yes | No | 25.5 | 2 | 1 | 9 | 50 |
| 17 | 75 | F | - | Recurrence | No | Yes | 37 | 3 | 2 | 37 | 89 |
| 18 | 80 | F | 3 | Primary | No | No | 13.5 | 1 | 0 | - | 47 |
| 19 | 72 | M | 2 | Primary | No | Yes | 37.0 | 1 | 4 | 21 | 120 |
| 20 | 75 | F | - | Primary | No | Yes | 18.8 | 1 | 4 | 11 | 148 |
| 21 | 75 | F | - | Recurrence | No | Yes | - | 1 | - | - | - |
| 22 | 67 | F | - | Primary | No | Yes | 38 | 1 | 0 | - | 117 |
| 23 | 51 | F | 4 | Recurrence | No | Yes | 18.1 | 1 | 1 | 72 | 168 |
| 24 | 73 | M | - | Recurrence | No | Yes | - | - | 9 | 5. | 168 |
| 25 | 80 | M | - | Primary | No | Yes | 27 | 1 | 3 | 70 | 136 |
| 26 | 50 | F | 2 | Recurrence | No | - | 11.5 | 1 | 8 | 108 | 180 |
| 27 | 81 | M | 3 | Recurrence | No | - | 8.0 | 1 | 4 | 98 | 123 |
| 28 | 49 | M | 2 | Recurrence | No | No | 5.8 | 1 | 2 | 129 | 175 |
| 29 | 58 | M | 3 | Primary | Yes | No | 9.4 | 3 | 4 | 203 | 268 |
| 30 | 78 | M | 3 | Recurrence | Yes | No | 9.8 | 3 | 6 | - | 268 |
| 31 | 82 | F | - | Recurrence | No | Yes | 15 | - | 5 | 108 | 333 |
| 32 | 90 | M | 3 | Primary | No | No | 79 | - | 3 | - | 112 |
| 33 | 73 | M | 2 | Recurrence | No | Yes | - | - | 8 | 18 | 168 |
