# Supplementary material for: Phosphorylation of IWS1 by AKT maintains liposarcoma tumor heterogeneity through preservation of cancer stem cell phenotypes and mesenchymal-epithelial plasticity
Source: Oncogenesis. 2023 May 26;12(1):30. doi: 10.1038/s41389-023-00469-z (PMC10219984; doi:10.1038/s41389-023-00469-z)
Supplement: Supplementary file 3 — Supplementary Table 2 [file 41389_2023_469_MOESM3_ESM.docx]

|  | Hazard ratio | Standard error | z | P value | [95% conf. interval] | |
| --- | --- | --- | --- | --- | --- | --- |
| logiws1 actin | 1.372 | 0.246 | 1.770 | 0.078 | 0.966 | 1.949 |
| logpiws1 actin | 1.338 | 0.194 | 2.010 | 0.045 | 1.007 | 1.777 |
| logpiws1 IWS1 | 1.170 | 0.242 | 0.760 | 0.448 | 0.780 | 1.756 |
