# Supplementary material for: Phosphorylation of IWS1 by AKT maintains liposarcoma tumor heterogeneity through preservation of cancer stem cell phenotypes and mesenchymal-epithelial plasticity
Source: Oncogenesis. 2023 May 26;12(1):30. doi: 10.1038/s41389-023-00469-z (PMC10219984; doi:10.1038/s41389-023-00469-z)
Supplement: Supplementary file 4 — Supplementary Table 3 [file 41389_2023_469_MOESM4_ESM.docx]

|  | Hazard ratio | Standard error | z | P value | [95% conf. interval] | |
| --- | --- | --- | --- | --- | --- | --- |
| logiws1 actin | 1.220 | 0.666 | 0.360 | 0.716 | 0.419 | 3.557 |
| logpiws1 actin | 1.439 | 0.832 | 0.630 | 0.529 | 0.463 | 4.470 |
| logpiws1 IWS1 | 2.925 | 2.822 | 1.110 | 0.266 | 0.441 | 19.377 |
