# Supplementary figures and images for: Phosphorylation of IWS1 by AKT maintains liposarcoma tumor heterogeneity through preservation of cancer stem cell phenotypes and mesenchymal-epithelial plasticity
Source: Oncogenesis. 2023 May 26;12(1):30. doi: 10.1038/s41389-023-00469-z (PMC10219984; doi:10.1038/s41389-023-00469-z)

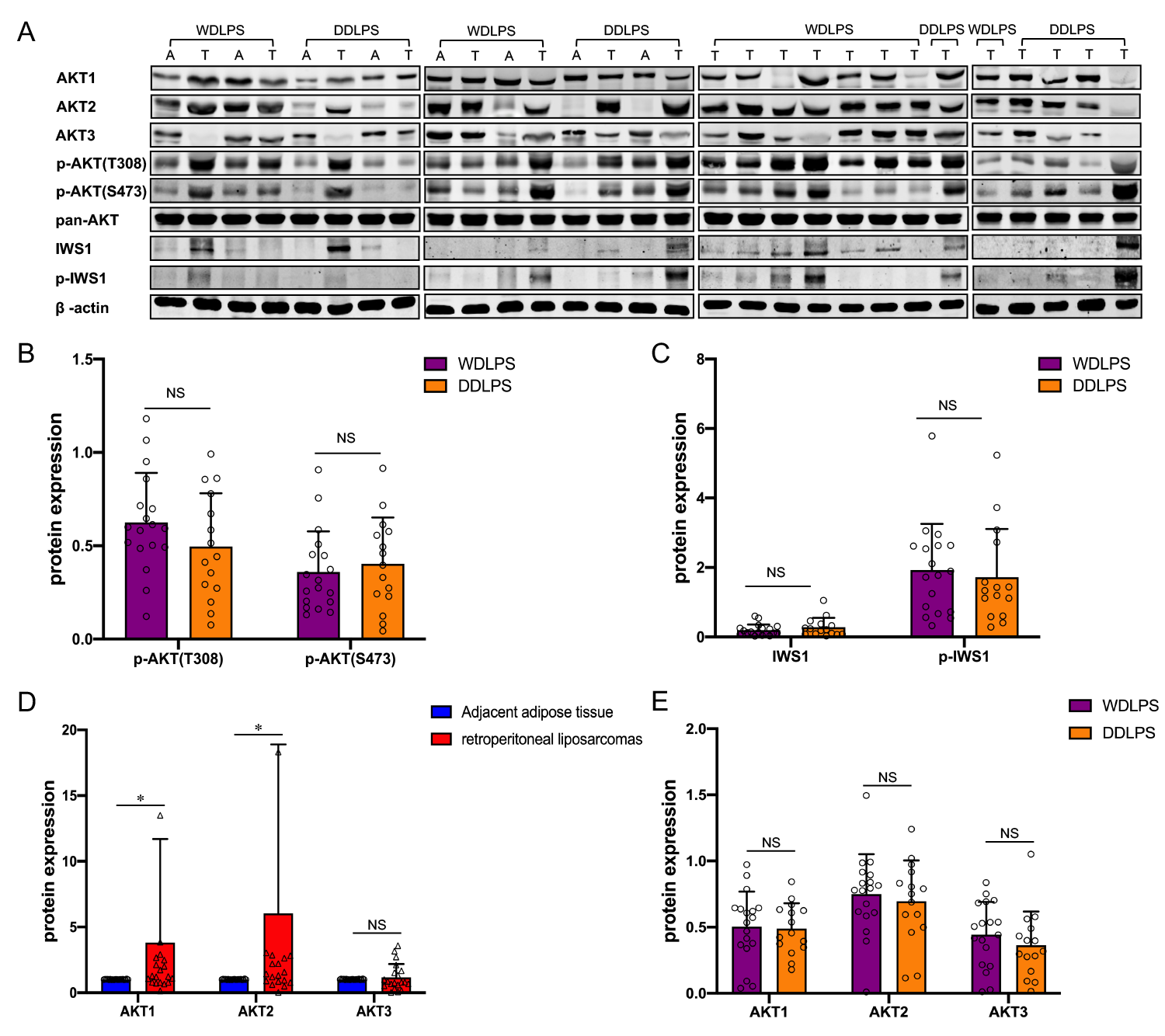

Supplement: Supplementary file 5 — Supplementary Figure 1 [file 41389_2023_469_MOESM5_ESM.tif]

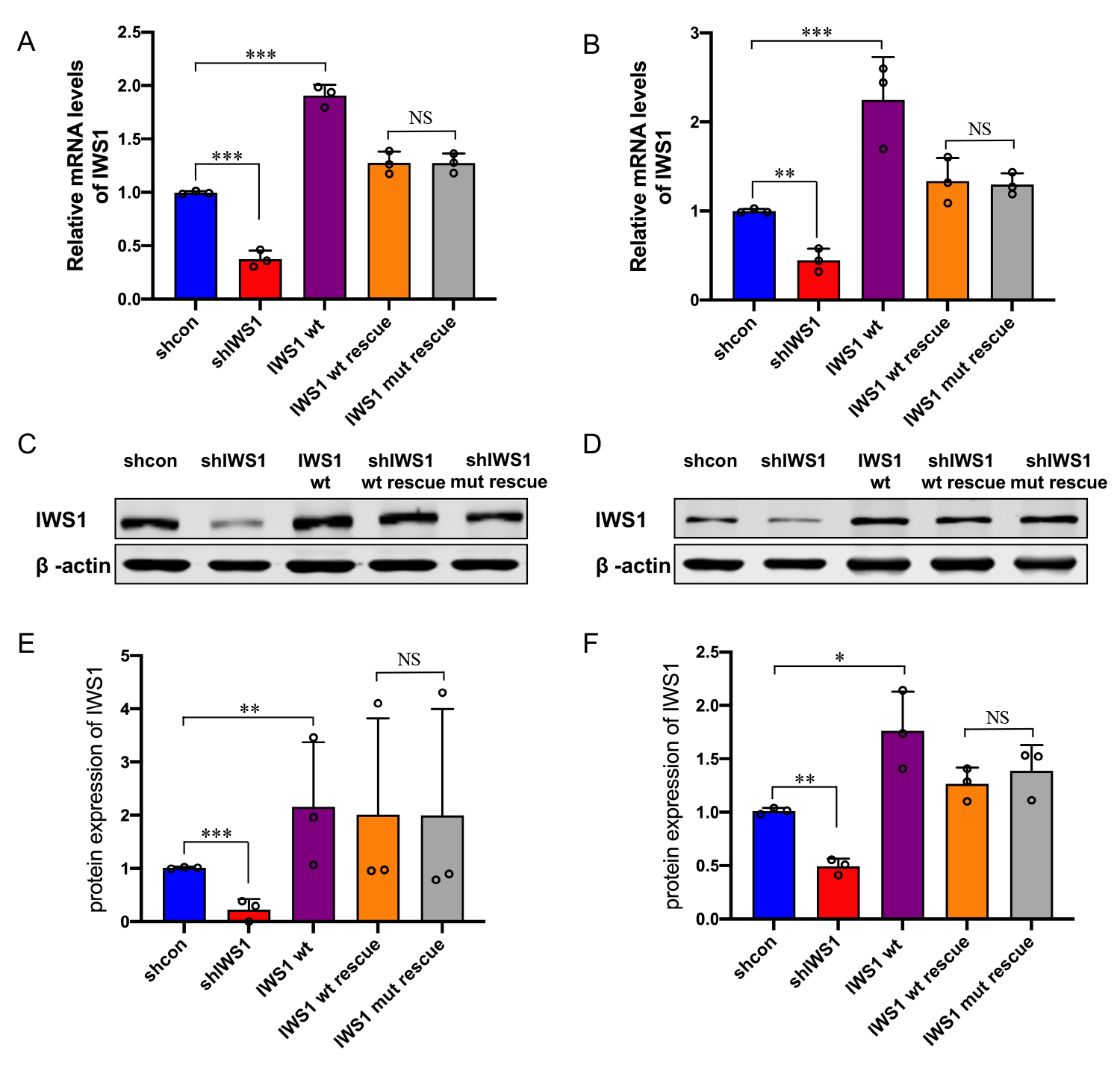

Supplement: Supplementary file 6 — Supplementary Figure 2 [file 41389_2023_469_MOESM6_ESM.tif]

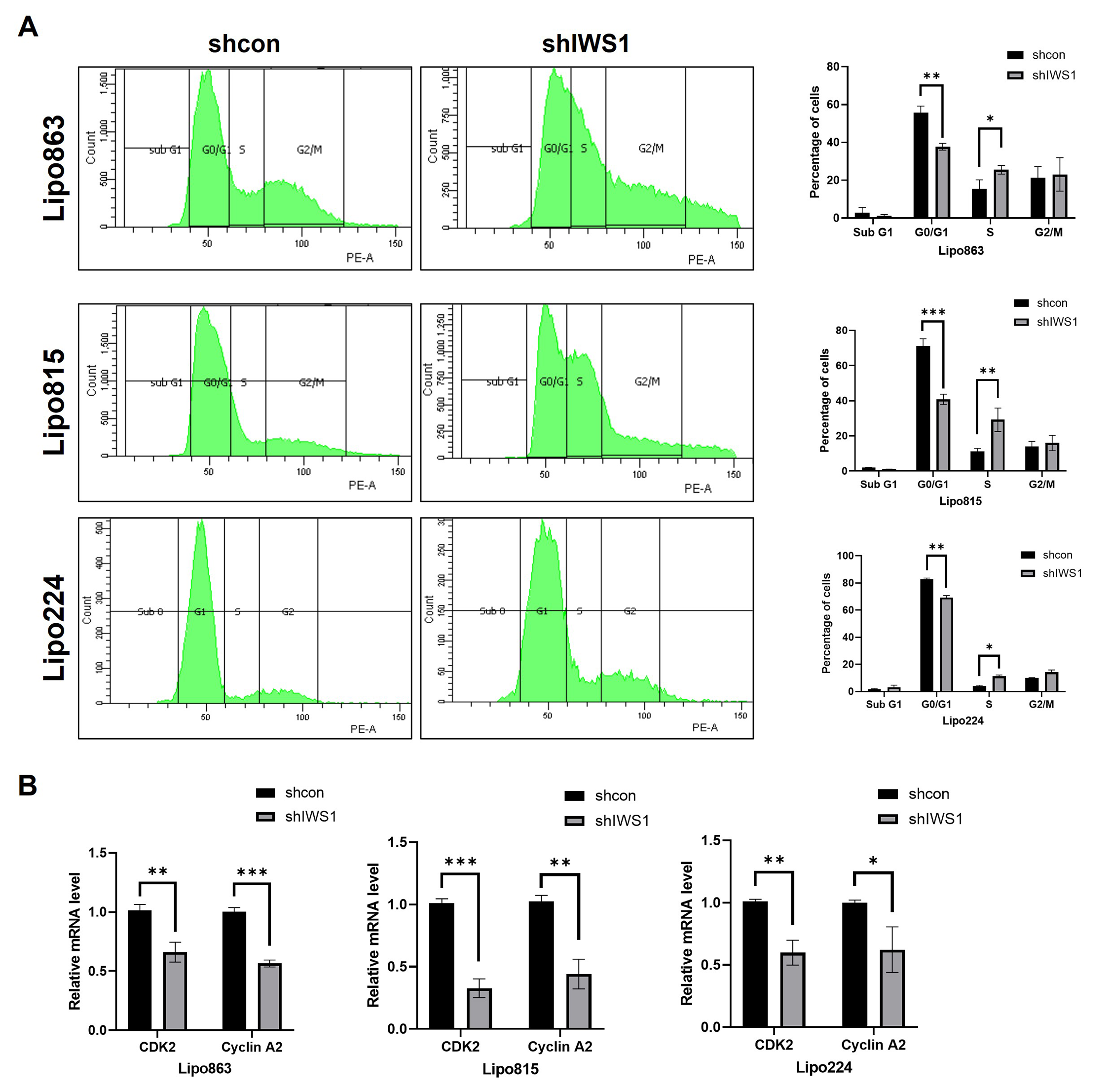

Supplement: Supplementary file 7 — Supplementary Figure 3 [file 41389_2023_469_MOESM7_ESM.tif]

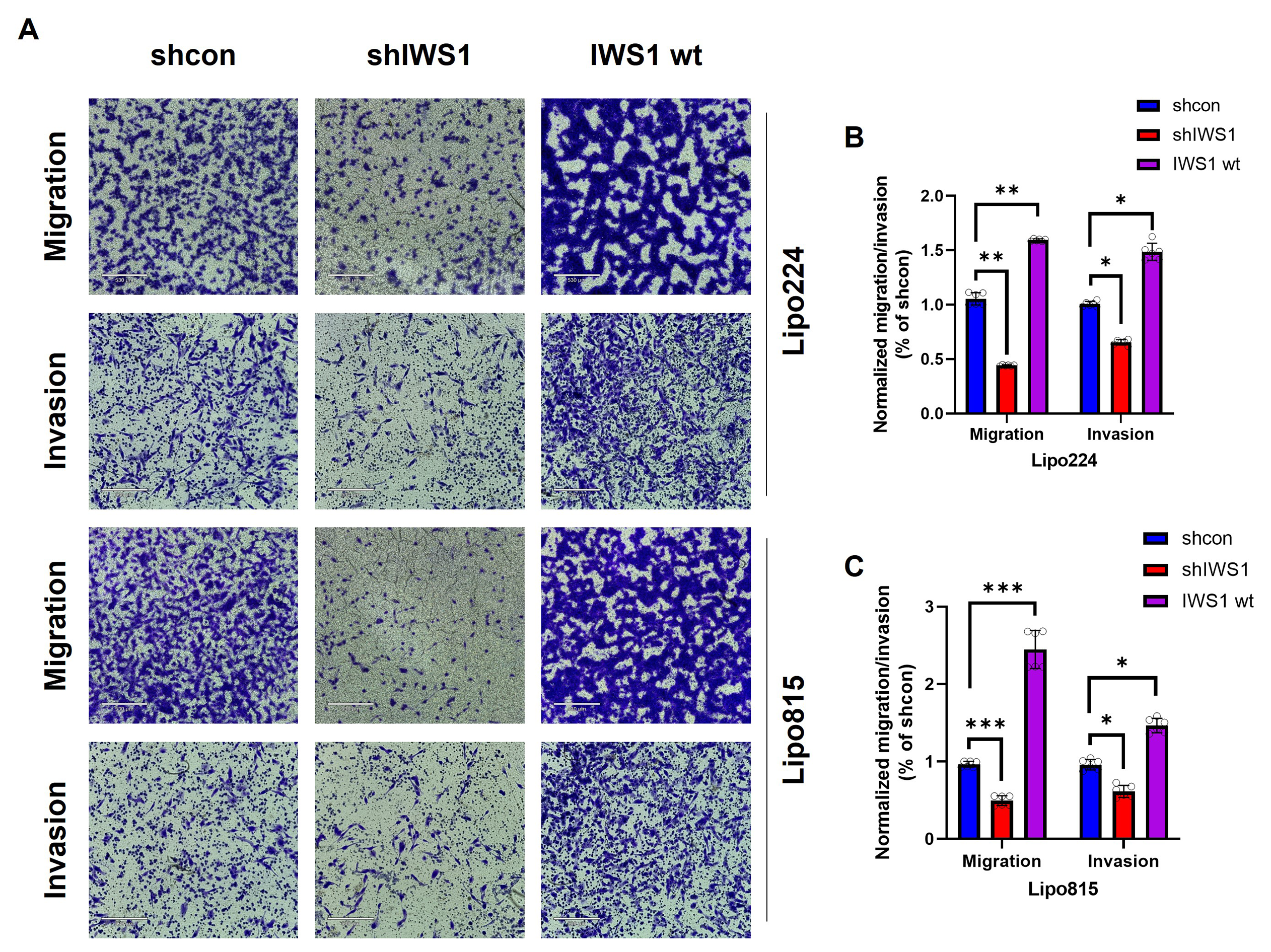

Supplement: Supplementary file 8 — Supplementary Figure 4 [file 41389_2023_469_MOESM8_ESM.tif]

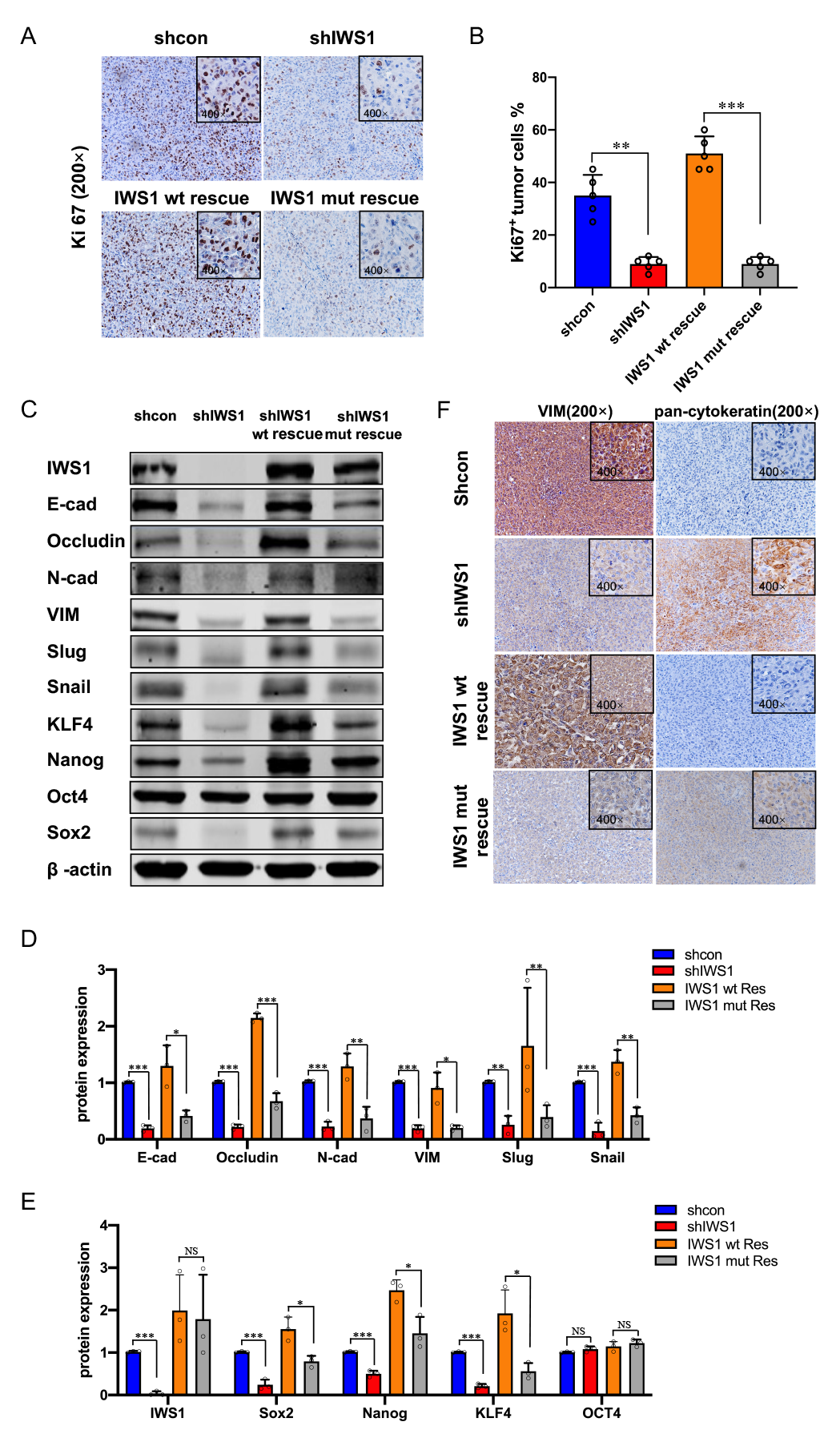

Supplement: Supplementary file 9 — Supplementary Figure 5 [file 41389_2023_469_MOESM9_ESM.tif]
